# How age of bilingual exposure can change the neural systems for language in the developing brain: A functional near infrared spectroscopy investigation of syntactic processing in monolingual and bilingual children^☆^

**DOI:** 10.1016/j.dcn.2013.06.005

**Published:** 2013-07-24

**Authors:** K.K. Jasinska, L.A. Petitto

**Affiliations:** a University of Toronto, Canada; b Gallaudet University, USA

**Keywords:** Bilingualism, Language development, Syntax, Sentence processing, fNIRS neuroimaging, Neural Signature Hypothesis

## Abstract

Is the developing bilingual brain fundamentally similar to the monolingual brain (e.g., neural resources supporting language and cognition)? Or, does early-life bilingual language experience change the brain? If so, how does age of first bilingual exposure impact neural activation for language?

We compared how typically-developing bilingual and monolingual children (ages 7–10) and adults recruit brain areas during sentence processing using functional Near Infrared Spectroscopy (fNIRS) brain imaging. Bilingual participants included early-exposed (bilingual exposure from birth) and later-exposed individuals (bilingual exposure between ages 4–6).

Both bilingual children and adults showed greater neural activation in left-hemisphere classic language areas, and additionally, right-hemisphere homologues (Right Superior Temporal Gyrus, Right Inferior Frontal Gyrus). However, important differences were observed between early-exposed and later-exposed bilinguals in their earliest-exposed language. Early bilingual exposure imparts fundamental changes to classic language areas instead of alterations to brain regions governing higher cognitive executive functions. However, age of first bilingual exposure does matter. Later-exposed bilinguals showed greater recruitment of the prefrontal cortex relative to early-exposed bilinguals and monolinguals.

The findings provide fascinating insight into the neural resources that facilitate bilingual language use and are discussed in terms of how early-life language experiences can modify the neural systems underlying human language processing.

## Highlights

• Early life bilingual language experience can change the developing brain.
• Age of first bilingual exposure predicts neural activation for language.
• Bilinguals show greater extent and variability of neural activity in language areas.
• Early-exposed bilinguals show greater activation in IFG and STG vs. monolinguals.
• Later-exposed bilinguals have greater DLPFC activity vs. early bilinguals.

## 1 Introduction

Early life experiences can have a profound impact on the developing brain and its organization (Newman et al., 2002, Ohnishi et al., 2001, Nelson, 2000, Greenough et al., 1987). Acting in unison, development and experience change the brain's physical structure and functional organization, allowing it to adapt to its environment (Hebb, 1949). Exposure to two languages early in life has the potential to yield structural changes in the brain (Mechelli et al., 2004) and changes in the patterns of neural activation during language processing within the brain's left hemisphere “classic language” areas and their right hemisphere homologues (Price, 2012, Gernsbacher and Kaschak, 2003), e.g., Left Inferior Frontal Gyrus (LIFG; Kovelman et al., 2008a, Heim et al., 2005, Caplan, 2001, Foundas et al., 1998) and Left Superior Temporal Gyrus (STG; Zatorre and Belin, 2001, Petitto et al., 2000). The left hemisphere dominance does not imply that the right hemisphere is not involved in language processing. For instance, the right hemisphere is involved in sentence processing and the integration of semantic information (Vigneau et al., 2011, Berl et al., 2010, Luke et al., 2002, Beeman et al., 2000).

Here, we ask whether the age of exposure to two languages would yield changes in the neural resources facilitating bilingual language use in the *developing* brain's language (LIFG; STG) and higher cognitive tissue (e.g., Dorsolateral Prefrontal Cortex; DLPFC; Balconi, 2013, Fuster, 2008, Petrides, 2005)? While the brain is undergoing maturational changes that support language development, would monolingual and bilingual children show *similar* or *different* patterns of behavior and neural activation when processing language, and would any differences be related to the age of bilingual exposure?

Direct comparisons of bilingual versus monolingual brains when processing language, specifically, during sentence processing tasks, gives insights into questions about whether monolinguals and bilinguals recruit the same brain areas, with the same extent and variability, and for the same linguistic functions. Extensive bilingual exposure results in both functional and structural changes in the person's brain (Wang et al., 2011, Kovelman et al., 2008a, Mechelli et al., 2004, Proverbio et al., 2002). Learning a second language has been found to increase the density of gray matter in the left inferior parietal cortex (Mechelli et al., 2004). Differential recruitment of classic language brain areas during a language processing task has been observed for bilingual relative to monolingual adults (Kovelman et al., 2008a). Using functional magnetic resonance imaging (fMRI), bilingual adults were found to recruit a greater extent and variability of the LIFG during a syntactic “sentence judgment task” relative to monolinguals. This increased recruitment of neural resources supporting linguistic processing in the bilingual brain revealed a “neural signature” of bilingualism; early exposure to two languages modifies the neural activation of neural sites and pathways that underlie language processing (Kovelman et al., 2008a). “Extent and variability” is a term previously published in neuroimaging and morphometry studies, both ours and others, to indicate the regions and areas encompassed in neural activation (e.g., Petitto et al., 2000, Penhune et al., 2003). Here, we refer to differences in the regions and areas of activation as “extent,” and differences in the increased or decreased activation as “variability,” thus, together, the “extent and variability.”

Remarkably, the degree of the neural changes is dependent on the age of acquisition and the proficiency levels in the second language (Klein et al., 2006, Perani et al., 1998). The focus on the age at which a young child is first exposed to another new family or community language has received much scientific attention over the past decade and a half within the discipline of Language Acquisition because of its importance to core issues such as the critical (and sensitive) period hypothesis (C/SPH) and the impact of different early experience on the developing brain. From Language Acquisition, research on children with dual language exposure within the first 3 years of life has been generally described as “early,” as in early-exposed bilinguals (Montrul, 2002, Meisel, 1989, Meisel, 1990, Meisel, 1994, Genesee et al., 1995, Paradis and Genesee, 1996, Paradis and Genesee, 1997, Hulk, 1997, Genesee, 2000, Kovelman et al., 2008b, Petitto et al., 2000); the literature has also referred to these children as “simultaneous bilinguals” (Kovelman et al., 2008b, Berens et al., 2013). By contrast, research on children with dual language exposure first beginning around ages 3–5 years has generally been described as “late,” as in late-exposed bilinguals (Klein et al., 2006, Montrul, 2002, Kovelman et al., 2008b, Petitto et al., 2000, Montrul, 2002, Vihman and McLaughlin, 1982, McLaughlin, 1984). This is because, remarkably, subtle differences in language forms (e.g., phonological and syntactic) have been observed when the new language exposure occurs after these ages (again, approximately 3–5 years; Kovelman et al., 2008b); the literature has also referred to these children as “sequential bilinguals” (Kovelman et al., 2008b, Berens et al., 2013). Thus, the “early” and “late” age benchmarks are the result of research findings investigating timing maturational and language acquisition milestones relative to important C/SPH and are the ones used in the present study.

Dual-language exposure in early life has the potential to mold neural organization and human language processing capacity, and there is substantial evidence that the age of second language exposure has both behavioral and neural implications for language processing. Early bilingual exposure yields high language competence outcomes (Johnson and Newport, 1989, Weber-Fox and Neville, 1996, Weber-Fox and Neville, 2001, McDonald, 2000, Kovelman et al., 2008b). Later second language acquisition impacts the attainment levels in the second language (Johnson and Newport, 1989, Lardiere, 1998, Montrul, 2009). It has been suggested that later exposure to a second language yields a different neural profile than early exposure with later-exposed bilinguals showing greater frontal and bilateral neural recruitment in the *second* language (Marian et al., 2003, Hahne and Friederici, 2001, Wartenburger et al., 2003). The level of language proficiency has also been found to impact the pattern of neural activity in the *second* language (Chee et al., 2004, Perani et al., 2003, Nauchi and Sakai, 2009); low-proficiency bilinguals demonstrate decreased activity in the LIFG relative to high-proficiency bilinguals for aspect of phonological and syntactic processing.

Certain levels of linguistic organization, critically phonology, aspects of morphology, and syntax, require exposure during key maturational age periods in order to achieve full behavioral mastery and native-like neural organization (Lenneberg, 1967). The present study examined aspects of syntactic processing in monolingual and bilingual children and adults. We conducted direct comparisons of the brains of early-exposed (birth to before age 3) bilinguals, later-exposed (age 3–6) bilinguals, and monolinguals using a sentence judgment task that participants performed while undergoing functional Near Infrared Spectroscopy (fNIRS) neuroimaging. fNIRS has significantly enhanced the ability to image human language and higher cognition; the technology provides excellent anatomical and temporal resolution, is quiet and “child-friendly”, and thus exceptionally suited for the study of language (see methods below for a more detailed description; see also Quaresima et al., 2012, for a review). Moreover, the present study provides a novel analytical advance. Here we applied complementary data analysis procedures, which, together, provide a powerful new view into the neural systems at the heart of human language processing.

Sentence judgment tasks have been widely used to assess grammatical knowledge in monolinguals and bilinguals (Kovelman et al., 2008a, Fernandez, 2002, Caplan, 2001). We used a relative-clause judgment task selected from a set previously used by Kovelman et al., 2008a (see also Caplan et al., 2008a, Caplan et al., 2008b, Caplan et al., 2003, Caplan et al., 2001, Caplan et al., 2000, Caplan et al., 1998, Caplan, 2001, Stromswold et al., 1996). All groups judged the semantic plausibility of an identical set of sentences in English. The specific sentences consisted of two types of syntactic complexity: the object-subject sentence type (OS, as in “The child spilled the juice that stained the rug”); and the subject-object sentence type (SO, as in “The juice that the child spilled stained the rug”). The object-subject sentence type is a right-branching relative clause construction, whereas the subject-object sentence type is a center-embedded relative construction. The “easier” right-branching relative clause sentence type is considered “unmarked” or “default” in English, whereas the “more difficult” center-embedded relative clause sentence type is considered to be “marked” (Stromswold et al., 1996).

This task has previously yielded highly consistent behavioral and neuroimaging results showing increased reaction times and inaccuracy, and increased neural activity in the LIFG for the SO sentence type relative to the OS sentence type (e.g., Kovelman et al., 2008a, Caplan et al., 2008a, Caplan et al., 2008b, Caplan et al., 2003, Caplan et al., 1998).

In the present study, we asked whether age of bilingual exposure can modify the neural organization in the brain's classic language areas (LIFG, STG) and cognitive areas (e.g., Dorsolateral Prefrontal Cortex; DLPFC) during sentence processing. Do monolingual, early-exposed and later-exposed bilingual children show *similar* or *different* patterns of behavior and neural activation?

Here we wondered whether the changes in neural activation of classic language tissue observed in adult bilinguals (called the “neural signature” for bilingualism; Kovelman et al., 2008a) occurs in children, and, if so, when is it revealed in the developing brain? Is the propensity for the expanded recruitment for language tissue developmentally malleable or fixed? How much depends on experience?

If young children are consistent with our adult findings (Kovelman et al., 2008a, Caplan et al., 2008a, Caplan et al., 2008b, Caplan et al., 2003, Caplan et al., 2000, Caplan et al., 1998), then, regarding sentence types, these child participants are predicted to show greater behavioral inaccuracy and reaction times for the more difficult SO sentence types relative to OS sentence types, with our adult controls performing faster and more accurately than the children. Regarding group differences, when tested in their earliest exposed language (i.e., English), we would predict that all three groups of children will show comparable accuracy and reaction times, that is, the young monolinguals, the early-exposed bilinguals, and the later-exposed bilinguals.

Behavioral results alone cannot reveal whether monolinguals, early-exposed and later-exposed bilinguals recruit the same brain areas, with the same extent and variability, and for the same aspects of sentence processing. Only neuroimaging yields testable hypotheses regarding what neural resources facilitate bilingual language use. Direct neuroimaging and behavioral comparisons of *developing* monolingual and bilingual brains can shed new light on the extent to which early life experiences can modify the neural systems underlying human language. A critical comparison, therefore, is whether monolinguals, early-exposed bilinguals, and later-exposed bilinguals’ behavior and pattern of neural activation during language processing is similar or different in their *first* language and earliest exposed language, in this case, English. Importantly, all participants had been exposed to English since birth (their L1), and were highly proficient, regular users of this language. Hence, in comparing monolinguals, early-exposed, and later-exposed bilinguals’ processing and neural activation in their first language, in contrast to a comparison between early-exposed and later-exposed bilinguals’ processing and patterns of neural activation in their *second* language, we could directly assess whether the age of bilingual exposure would yield changes in neural organization supporting sentence processing in the native language of all participants.

We examined the hypothesis that bilingual exposure reflects more robust neural activity; hence, a “neural signature” of bilingualism (Kovelman et al., 2008a), and this increased recruitment of neural resources supporting linguistic processing in the bilingual brain varies with the age of bilingual exposure. That is, early and later-exposed bilinguals show differential recruitment of neural resources for syntactic processing. Later bilingual exposure reflects more robust cognitive-general activity (greater DLPFC recruitment), relative to both monolingualism and early bilingualism.

We have previously observed an increased extent and variability in neural recruitment among Spanish-English bilingual adults as compared with English monolingual adults (Kovelman et al., 2008a). Here we test the hypothesis that bilingual exposure recruits a greater extent and variability of the neural resources for language processing (which would constitute the neural signature of bilingualism) in the adult bilingual brain. We further predict that this neural signature of bilingualism will be observed among other adult bilingual language pairs such as English-French, among others.

If the developing bilingual brain shows greater neural activation in classic language tissue, this would lend support to the “neural signature” hypothesis and reveal its origins in development. That is, early bilingual language exposure modifies neural activation in language-dedicated neural sites and pathways before they are fully matured. Furthermore, if early-exposed and later-exposed bilinguals show *different* patterns of neural activity for language processing in their first language, this would indicate that bilingualism abides by principles of “Sensitive Period Hypothesis” (Lenneberg, 1967, Newport, 1990), and crucially, that the age of dual-language exposure has the potential to yield changes in neural activation supporting all language processing, and not only second language processing. Additionally, in correspondence with the behavioral predictions above, we predict that both children's and adults’ neuroimaging data to show greater neural activation in the LIFG for the more difficult SO sentence types relative to OS sentence types.

The present study further permits us a novel lens into a prevailing controversy in the field involving the fundamental nature of language in *the bilingual brain*. If bilingualism is largely a language-specific activity, then bilinguals should demonstrate more robust neural activity in classic left hemisphere language areas such as the LIFG and STG relative to monolinguals. By contrast, if bilingualism is largely a cognitive-general activity, then the dual language processing demands placed on executive functions (such as, attention) may yield more robust neural activity in the DLPFC in bilinguals relative to monolinguals.

## 2 Methods

### 2.1 Participants

20 bilingual children (9 female and 11 male, mean age = 8.9, range = 7–10 years) and 20 English monolingual children (13 female and 7 male, mean age = 8.92, range = 7–10 years) along with 10 bilingual adults (7 female and 3 male, mean age = 19.9, range = 17–26 years) and 9 English monolingual adults (8 female and 1 male, mean age = 19, range = 17–24 years) participated in the study. We excluded one English monolingual adult from the study during offline data analysis as their fNIRS signal was unreliable. Bilingual participants were further divided into two groups: early (3 female and 7 male, mean age = 8.9, range = 7–10 years) and later exposed (6 female and 4 male, mean age = 9, range = 7–10 years). 10 bilingual child and 10 bilingual adult participants received simultaneous exposure to their two languages from birth (early exposed bilinguals). 10 bilingual child participants received exposure to their second language between the ages of 4–6 (later exposed bilinguals) (see the summary of participant information in Table 1).

**Table 1:** Participant information.

Group/age	Age at testing	Age of first exposure to	
	Mean	English	Other language
Monolinguals			
Children	8.9	Birth	n/a
Adults	19	Birth	n/a
Early bilinguals			
Children	8.9	Birth	Birth
Adults	19.9	Birth	Birth
Late bilinguals			
Children	9	Birth	4–6

All participants were native speakers of English and had begun acquiring this language from birth. Most of the bilingual participants (13 out of 20 children and 2 out of 10 adults) were English-French bilinguals. As a specific design feature of this study, the remaining bilingual participants spoke languages from a varied linguistic pool. These languages included Cantonese, Vietnamese, Tagalog, Tamil, Arabic, Urdu, Punjabi, Spanish, Russian, German and Greek.

We specifically selected bilingual participants that would yield language pairs from topologically distinct languages covering analytical languages (e.g., English), morphologically rich languages (e.g., Russian, Spanish, Urdu), different writing systems (e.g., Cyrillic), and word orders (e.g., SVO (German), VSO (Arabic)). In doing so, we could directly compare monolingual versus bilingual brains. If we had only compared bilinguals with one language pairing (e.g., English-French) to English monolinguals, we may have observed differences that could be attributable to differences specifically between English-French bilinguals and English monolinguals. We could not be sure that we have observed differences generalizable to all bilinguals versus all monolinguals and not only to English speakers versus English-French speakers. That is, differences between groups could reflect difference between monolinguals and bilinguals, or, alternatively linguistic differences between, for example, English and French. Perhaps, some feature of the French language in contrast with the English language could yield different neural patterns of activation. Thus, our study design controlled for these potential confounds.

All children were similar in socio-economic status (SES) as indexed by maternal education and occupation (Vekiri, 2010, SéAguin et al., 1999). SES was coded on a scale of one through four based on the following grouping: upper-SES = professionals with “college graduate”, upper-middle-SES = service sector workers with “college graduate”, middle-SES = service sector with “high school/GED” and “blue-collar workers” with “college graduate”, and lower-SES = blue collar workers with “high school/GED”. Mean SES rank for monolingual children was 3.4, mean SES rank for early-exposed bilingual children was 3.4 and mean SES rank for later-exposed bilingual children was 3.0 (*F*(1,25) = .185, *p* > .05, ns).

Exclusion criteria for participants consisted of speech/language disorders, reading disabilities, developmental delays, or any other neurological condition. Children with significant vision or hearing problems that would interfere with their ability to participate were also excluded. Exclusion criteria, vision and/or hearing problems were self-reported by participants, or reported by parents of participants. All participants were right-handed. All participants were living in Toronto, Canada at the time of testing. The parents received monetary compensation for their travel and adults received credit toward their first-year psychology course for their participation in the study. This study received ethical approval from the research ethics review board at the University of Toronto.

#### 2.1.1 Participant screening

##### 2.1.1.1 Assessment of Bilingual Language Background and Use

Parents and adult participants filled in a highly detailed standardized, previously validated and published questionnaire, which contained cross-referenced questions (internal validity questions) called the “Bilingual Language Background and Use Questionnaire” (“BLBUQ;” see Holowka et al., 2002, Kovelman et al., 2008a, Kovelman et al., 2008b, Petitto et al., 2001, Petitto et al., 2000, Penhune et al., 2003 for more details on this extensive bilingual language questionnaire). Participants were grouped as monolinguals, early-exposed or later-exposed bilinguals based on the age of first bilingual exposure. All participants and parents of child participants reported high language proficiency and language use in English and in their second language. Proficiency and use was based on reported input languages of parents, the languages used in the home and at school, and the relative amount of exposure in each language throughout their lives on the Bilingual Language Background and Use Questionnaire. This questionnaire asked (a) detailed questions about parents’ language use and attitudes (language background, educational history, employment facts, social contexts across which each parent uses his or her languages, personal language preference containing standardized questions to assess language dominance and language preference, personal attitudes about language/s, language use with the child and participant's other siblings, parents’ linguistic expectations for their child, parents’ attitudes toward bilingualism, parents’ self-assessment about “balanced” bilingual input, and (b) detailed questions about the nature of language input and use with the child languages used with the child, questions about child rearing, questions about who cares for the child and number of hours, caretaker's language/s, child's exposure patterns to television/radio).

An important feature in the design of any study, particularly studies of neural processing, is the requirement that there be strong confidence in one's experimental and control groups. While the participants were indeed grouped as to their age of first bilingual language exposure (that is, “early-exposed” versus “late-exposed”), extreme rigor is applied to achieve cross-confirmation for group inclusion, which is inclusive of such factors as the participants’ dual language proficiency, language use, input languages of their parents, and the like. To ensure this cross-confirmation rigor, such information is assessed through our use of the standardized and previously validated assessment tool, Bilingual Language Background and Use Questionnaire.

### 2.2 Stimuli

While undergoing fNIRS neuroimaging, participants were presented with the sentence judgment task. Both children and adults were presented with 64 English sentences. The set of sentences has previously been used by Kovelman, Baker and Petitto (2008a), which were provided directly to this research team by David Caplan (Caplan et al., 2008a, Caplan et al., 2008b, Caplan et al., 2003, Caplan et al., 2000, Caplan et al., 1998, Stromswold et al., 1996). Sentences were divided into conditions based on the OS/SO syntactic distinction, and further divided into plausible and implausible. Thus, the task was comprised of four conditions: OS plausible (*The light-house guided the sailor that piloted the boat*), OS implausible (*The sailor guided the light-house that piloted the boat), SO plausible (*The sailor that the light-house guided piloted the boat*) and SO implausible (*The light-house that the sailor guided piloted the boat). In the plausible OS construction, the head of the relative clause (*the light-house*) is the object of the main clause and the subject of the verb of the relative clause. In the plausible SO construction, the head of the relative clause (*the sailor*) is the subject of the main clause and the object of the verb of the relative clause (Chen et al., 2006).

A number of considerations were made when the stimuli were designed (Stromswold et al., 1996). The sentences are based on scenarios, with each scenario appearing equally often across each condition. Thus, differences in semantics, word frequency, word choice could not be responsible for differences in hemodynamic response across sentence types. The animacy of subject and object noun phrases and sentence plausibility varied orthogonally, thus, subjects could not use the sequence of animacy in order to make plausibility judgements. All noun phrases were singular, common and definite. Sentences became implausible at various points such that participants had to read the entire sentence before making a judgment.

In this study, we sought to examine differences among monolinguals, early- and later-exposed bilinguals during syntactic processing. Our data analysis and results (below) apply only to the plausible OS and SO sentence types. The focus of the current study on the syntactic distinction between OS and SO sentences is theoretically motivated. We specifically selected the syntactic complexity distinction among OS and SO sentences as it permitted us to examine an aspect of language structure, syntax, which requires linguistic experience during key maturational ages for acquisition. An additional design feature in using the OS/SO distinction is that, beyond syntax, it provides insight into whether the brain might reveal different neural patterns of activation for different types of syntactic constructions. It also reveals whether these more subtle syntactic distinctions are differentially impacted by early bilingual experience.

### 2.3 Procedure

We used a Dell computer running E-prime software to present the stimuli and record behavioral responses (response latencies and judgment accuracy). The participants were seated approx 30 cm from the stimulus presentation monitor. This study utilized an event-related method, with counterbalanced presentation of each sentence type. Duration of stimulus presentation varied with participants’ response latency. Participants were instructed to judge the plausibility of each sentence with a button press. Crucially, participants were instructed to use only their right index finger to make their selection in order to keep motor neural activity consistent across all conditions. After participants’ button press, a fixation cross was displayed for 2 s. The entire experiment was approximately 20 min.

### 2.4 fNIRS brain imaging

#### 2.4.1 fNIRS advantages over fMRI

Like fMRI, fNIRS measures changes in blood oxygenation levels, but has several critical advantages over fMRI. fNIRS has a sampling rate of 10 Hz, as compared to fMRIs sampling rate of ∼once every 2 s. Thus, fNIRS is regarded as a closer measure of neural activity than the fMRI. Unlike fMRI that yields a combined blood oxygen level density (BOLD) measure (a ratio between oxygenated and deoxygenated hemoglobin), fNIRS yields separate measures of deoxygenated and oxygenated hemoglobin in “real time” during recording. fNIRS has good spatial resolution and it has better temporal resolution than fMRI (∼<5 s hemodynamic response, HR). fNIRS’ depth of recording is about ∼3–4 cm deep, but this is well-suited for studying the brain's higher cortical functions, such as language. Perhaps fNIRS’ greatest advantage over fMRI for cognitive neuroscience research with humans is that it is very small (the size of a desktop computer), portable, and virtually silent. This latter feature makes it outstanding for testing natural language processing, as the ambient noise typical of the fMRI scanner are not present. Other important advantages over fMRI is that fNIRS is especially participant/child friendly (adults and children sit normally in a comfortable chair), and, crucially, it tolerates moderate movement. In summary, it is the fNIRS’ capacity to provide information on changes in blood oxygen level densities/BOLD (including total, oxygenated, and deoxygenated hemodynamic change), its high sampling rate, relative silence, higher motion tolerance than other systems, and child friendly set-up, have lead to the growing use of fNIRS as one of today's leading brain imaging technologies.

#### 2.4.2 fNIRS data acquisition

**Fig. 1:**
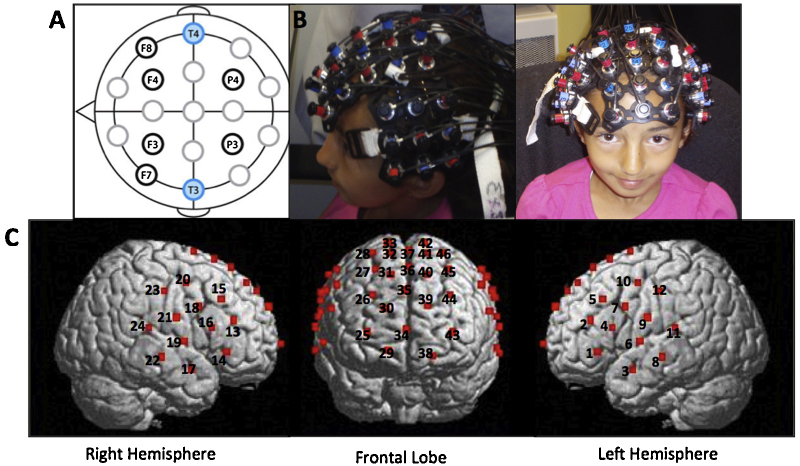
(a) fNIRS placement (a) key locations in Jasper (1958) 10–20 system. The detector in the lowest row of optodes was placed over T3/T4; (b) Probe arrays were placed over left-hemisphere language areas and their right-hemisphere homologues as well as the frontal cortex. (c) Location of 46 channels.

The hemodynamic response was measured with a Hitachi ETG-4000 Near Infrared Spectroscopy system with 46 channels, acquiring data at 10 Hz. The lasers were factory set to 690 and 830 nm. The 18 lasers and 15 detectors were segregated into one 3 × 5 array and two 3 × 3 arrays (see Fig. 1a and b). Once the participant was comfortably seated, one array was placed on each side of the participant's head and one array was placed over top. Positioning of the array was accomplished using the 10–20 system (Jasper, 1958) to maximally overlay regions classically involved in language areas in the left hemisphere as well as their homologues in the right hemisphere, and attentional and executive functioning areas in the frontal lobe (for additional details, and prior fMRI–fNIRS co-registration procedures to establish neuroanatomical precision of probe placements, see Petitto et al., 2012, Kovelman et al., 2008c, Kovelman et al., 2008d).

Prior to recording, every channel was tested for optimal connectivity (signal/noise ratio) using Hitachi fNIRS inbuilt software. Digital photographs of left, right, front and top views were also taken of the positioning of the probe arrays on the participants’ head prior to and after the recording session to ensure that probes remained in their identical and anatomically correct pre-testing placement. The Hitachi system collects MPEG video recordings of participants simultaneous with brain recording, which is synchronized with the testing session. This outstanding feature, among other things, makes possible the identification of any larger movement artifacts that may have impacted a select channel which then be identified during offline analysis.

### 2.5 Analysis

#### 2.5.1 Behavioral analysis

We first asked whether monolinguals and bilinguals show different or similar latencies and accuracy rates across sentence types (SO and OS). Next, we asked whether performance varied with the participant's age (Child or Adult) and the age at which the participant acquired their second language (L2 AoA: Early and Late). We performed a 2 × 2 × 2 × 2 (Language Group × Sentence Type × Age × L2 AoA) linear mixed effects model incorporating crossed random effects (i.e., random intercepts) of subjects.

#### 2.5.2 fNIRS data pre-processing and analysis

After the neuroimaging recording session, data were exported and then analyzed using a novel analytical procedure whereupon two statistical techniques were applied: *statistical parametric mapping* and *multilevel modeling* of changes in HbO concentrations. By doing so, we increased the statistical inferences afforded by each individual technique, thereby rendering more powerful the insights possible from our neuroimaging data. In addition, the use of these two different statistical techniques permitted us the ability to corroborate the findings across the statistical approaches, and therefore enabled us to increase the reliability of our results.

First, data were analyzed using a Matlab-based statistical software package: Statistical Parametric Mapping for NIRS (NIRS-SPM, Version 3.1) (Ye et al., 2009, Jang et al., 2009). Using the modified Beer–Lambert equation, NIRS-SPM converts optical density values into concentration changes in oxygenated and deoxygenated hemoglobin response (HbO and HbR, respectively). Changes in HbO and HbR concentrations were filtered with a Gaussian filter and decomposed using a Wavelet-Minimum Description Length (MDL) detrending algorithm in order to remove global trends resulting from breathing, blood pressure variation, vasomotion, or participant movement artifacts and improve the signal-to-noise ratio (Jang et al., 2009). NIRS-SPM allows the spatial registration of NIRS channels to MNI space without structural MRI (Singh et al., 2005) by using a three dimensional digitizer (Polhemus Corp.) and provides activation maps of HbO, HbR and THb based on the general linear model and Sun's tube formula correction (Sun, 1993, Sun and Loader, 1994). The HbO values were used in all subsequent analyses (for detailed methods see especially, Kovelman et al., 2009, Shalinsky et al., 2009).

Secondly, a multilevel modeling (MLM) framework was also used in this study. The linear mixed model has been shown to decrease the likelihood of committing both Type I and II errors versus ANOVA (Baayen et al., 2008, Locker et al., 2007). The data were analyzed as a two level random intercept variance component model where the dependent variable represented change in HbO concentration using the statistical software package SPSS (IMB Inc.). First, a null model comprising individual NIRS channels (level 1) nested in participants (level 2) with no predictor variables was computed. In the case of the null model, the significance of the random term indicates between subject and channel variation in HbO concentration changes. Next, the null model was expanded to include fixed effects for Sentence Type, Language Group, Age, Hemisphere, Age of Second Language Acquisition, as well as interaction terms. The improvements in the fit of the full model over the null model were assessed using the Log Likelihood statistic (*χ*^2^(1, *N* = 19) = 700.03, *p* = .05).

In this study, one innovation was the application of two different but complementary data analyses approaches (SPM and MLM analyses). SPM analyses test differences in neural activation at specific sites, that is, a series of univariate *t*-tests for each fNIRS channel (or, for example, if this were an fMRI study, the fMRI voxel). By contrast, MLM analyses afford us the ability to incorporate the ROI as a variable in the model. As such, our MLM analysis permitted us to model different hemodynamic response patterns brain-wide by incorporating the hierarchy of data structure. The significant advance that a combination of SPM and MLM analyses gives us over an SPM-only approach is that we can simultaneously model neural activation across all ROIs, and thus, do not need to correct for separate, multiple contrasts.

Neuroimaging analyses were divided into whole brain and ROI analyses. Our ROI analyses were conducted individually for children and adults at left and right IFG, left and right STG, DLPFC, and Primary Motor Cortex (control site).

#### 2.5.3 Whole-Brain all Channel analysis

We began with whole brain analyses and asked whether monolingual and bilingual children show different or similar patterns of neural activity across sentence types (SO and OS), as well as whether the pattern of neural activity varied among early-exposed and later-exposed bilingual children. Next, we asked whether the pattern of neural activity observed in monolingual and bilingual children would be similar to or different from the pattern of neural activity observed in monolingual and bilingual adults. To do this, we first generated *t*-statistic HbO activation maps with left hemisphere, right hemisphere and frontal views comparing Language Groups (Monolingual and Bilingual), Sentence Type (OS and SO), and L2 AoA (Early-exposed or Later-exposed). Second, we performed a 2 × 2 × 2 (Language Group × Sentence Type × L2 AoA) multilevel model (MLM) of changes in HbO concentration for child participants and a 2 × 2 (Language Group × Sentence Type) multilevel model (MLM) of changes in HbO concentration for adult participants across all channels. Values representing concentration changes in HbO were nested within channels, which were nested within each participant. Thus, our model incorporated crossed random effects (i.e., random intercepts) of participants and individual NIRS channels.

#### 2.5.4 Identifying Regions of Interest (ROI) for further analysis

In the 3 × 3 and 3 × 5 recording arrays, channels are defined as the area between adjacent lasers and detectors. Each channel is comprised of two attenuation values from the 690 nm and 830 nm lasers, as per settings of the ETG-4000 system. Attenuation values from each channel were converted to HbO and HbR values using the Modified Beer-Lambert equation (Shalinsky et al., 2009, Ye et al., 2009, Jang et al., 2009). Thus, the channels referred to changes in HbO and HbR concentrations in the regions between lasers and detectors. We performed spatial registration of NIRS channels to MNI space using MNI coordinates from available SPM template images (Ye et al., 2009, Jang et al., 2009). The spatial registration yielded values for Brodmann areas maximally represented by each channel, which guided the selection of ROIs. As an added measure, we selected channels over the Primary Motor Cortex that did not correspond to areas of language or higher cognitive processing as control sites.

PCA analyses: as an added measure, we also performed a PCA to identify clusters of channels with robust activity. From these PCA results, we matched these channels to the corresponding Brodmann areas to validate our ROI selection. 12 components emerged from the principal component analysis, of which the first four accounted for 45.3% of the total variance. Channels corresponding to region of the frontal lobe including the DLPFC were most correlated with the first component, bilateral STG channels were most correlated with the second component, LIFG channels were most correlated with the third component, and left posterior STG channels were most correlated with the fourth component. Thus, our ROIs included the brain's classic language processing areas, especially channels maximally overlaying the LIFG (BA 45/47; Broca's area 44/45) and the STG (BA 42/22), channels maximally overlaying the DLPFC (BA 9/46), as well as a control site.

#### 2.5.5 Regions of Interest (ROI) analysis

We performed a 2 × 2 × 2 (Language Group × Sentence Type × L2 AoA) multilevel model (MLM) of changes in HbO concentration for child participants and a 2 × 2 (Language Group × Sentence Type) multilevel model (MLM) of changes in HbO concentration for adult participants across six brain regions: left IFG, right IFG, left STG, right STG, DLPFC and Primary Motor Cortex (control site).

## 3 Results

### 3.1 Behavioral results

#### 3.1.1 Reaction time

This analysis revealed a main effect of sentence type (OS and SO; *F*(1,67) = 33.353, *p* < .001) and a main effect of age (Child and Adult; *F*(1,67) = 11.839, *p* < .001). There was no main effect of language group (Monolingual and Bilingual; *F*(1,67) = .174, *p* > .05) or L2 AoA (Early-exposed or Later-exposed; *F*(1,91) .316, *p* > .05). Overall, all participants demonstrated significantly faster latencies for OS than SO sentences, and adults demonstrated significantly faster latencies than children (see Table 2).

**Table 2:** Monolinguals and bilinguals performed more rapidly and more accurately on OS sentences than SO sentences.

Group/age	RT (ms)		% Correct	
	Mean (SD)		Mean (SD)	
	SO	OS	SO	OS
Monolinguals	5988 (436)	4956 (435)	56.7 (3.7)	75.5 (3.7)
Children	6969 (419)	6123 (418)	49.5 (3.6)	63.5 (3.6)
Adults	5006 (766)	3788 (764)	64.0 (6.6)	87.5 (6.6)
Early bilinguals	6088 (420)	5347 (420)	62.9 (3.6)	82.3 (3.6)
Children	6964 (519)	6378 (518)	56.1 (4.4)	74.6 (4.4)
Adults	5212 (661)	4316 (662)	69.6 (5.6)	90.0 (5.6)
Late bilinguals				
Children	6547 (713)	5831 (712)	63.1 (6.2)	69.3 (6.2)

#### 3.1.2 Accuracy

This analysis also revealed a main effect of sentence type (OS and SO; *F*(1,67) = 29.776, *p* < .001), a main effect of age (Child and Adult; *F*(1,67) = 13.958, *p* < .001). There was no main effect of language group (Monolingual and Bilingual; *F*(1,67) = 2.064, *p* > .05) or L2 AoA (Early-exposed or Later-exposed; *F*(1,67) = .017, *p* > .05). Overall, all participants demonstrated significantly higher accuracy rates on OS than SO sentences, and adults demonstrated significantly higher accuracy rates than children (see Table 2).

### 3.2 Neuroimaging results

Main effects of Sentence Type, Language Group, and Age of Second Language Exposure across all channels and at ROIs are summarized in Table 3.

**Table 3:** Main effects of Sentence Type, Language Group, and Age of Second Language Exposure across all channels and at ROIs.

	Sentence Type	Language Group	Age of Second Language Exposure
*Whole-Brain all Channel*			
Children	SO > OS *F*(1,3715444) = 4.472, *p* < .05	Bilinguals > Monolinguals *F*(1,1701) = 4.464, *p* < .05	Later-Exposed > Early-Exposed *F*(1,1701) = 4.464, *p* < .001
Adults	SO > OS *F*(1,1267769) = 115.882, *p* < .001	Bilinguals > Monolinguals *F*(1,851) = 8.351, *p* < .01	N/A
*Left Inferior Frontal Gyrus*			
Children	SO > OS *F*(1,157185) = 24.407, *p* < .001	Non-sig. *F*(1,70) = .180, *p* > .05	Later-Exposed > Early-Exposed *F*(1,157186) = 269.702, *p* < .001
Adults	SO > OS *F*(1,44109) = 25.158, *p* < .001	Non-sig. *F*(1,31) = .655, *p* > .05	N/A
*Right Inferior Frontal Gyrus*			
Children	SO > OS *F*(1,131688) = 36.179, *p* < .001	Non-sig. *F*(1,57) = .180, *p* > .05	Non-sig. *F*(1,57) = .007, *p* > .05
Adults	SO > OS *F*(1,56322) = 125.458, *p* < .001	Non-sig. *F*(1,31) = .655, *p* > .05	N/A
*Left Superior Temporal Gyrus*			
Children	SO > OS *F*(1,125084) = 18.068, *p* < .001	Non-sig. *F*(1,57) = .050, *p* > .05	Later-Exposed > Early-Exposed *F*(1,57) = 8.992, *p* < .01
Adults	SO > OS *F*(1,65128) = 124.052, *p* < .001	Non-sig. *F*(1,43) = .846, *p* > .05	N/A
*Right Superior Temporal Gyrus*			
Children	SO > OS *F*(1,185108) = 27.417, *p* < .001	Bilinguals > monolinguals *F*(1,82) = 4.170, *p* < .05	Later-Exposed > Early-Exposed *F*(1,82) = 4.556, *p* < .05
Adults	Non-sig. *F*(1,70921) = .122, *p* > .05	Bilinguals > monolinguals *F*(1,46) = 3.145, *p* = .083	N/A
*Dorsolateral Prefrontal Cortex*			
Children	SO > OS *F*(1,636222) = 7.983, *p* < .01	Non-sig. *F*(1,287) = 2.579, *p* > .05	Later-Exposed > Early-Exposed *F*(1,287) = 20.121, *p* < .001
Adults	SO > OS *F*(1,160885) = 20.666, *p* < .001	Non-sig. *F*(1,102) = 2.068, *p* > .05	N/A
*Control site (Primary Motor Cortex)*			
Children	Non-sig. *F*(1,821388) = .000, *p* > .05	Non-sig. *F*(1,376) = .192, *p* > .05	Non-sig. *F*(1,376) = 2.553, *p* > .05
Adults	Non-sig. *F*(1,269369) = .451, *p* > .05	Non-sig. *F*(1,177) = 3.040, *p* > .05	N/A

#### 3.2.1 Whole-Brain all Channel

##### 3.2.1.1 Children

**Fig. 2:**
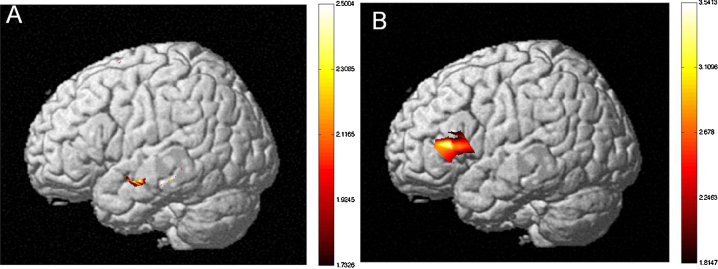
Neural activations for SO relative to OS sentence types for (a) child participants and (b) adult participants (*t*-statistic map from HbO, *p* = .05, corrected). Participants showed increased neural activity in the left hemisphere SO sentence types relative to OS sentence types. Children showed increased neural activity in the temporal lobe (MTG) and adults showed increased neural activity in the LIFG.

**Fig. 3:**
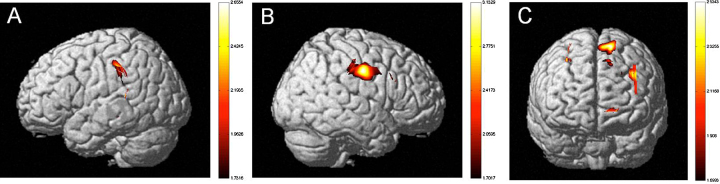
Neural activation of early-exposed bilingual children as compared to monolingual children (*t*-statistic map from HbO, *p* = .05, corrected). Early-exposed bilingual children show more robust neural activation in (a) the left and (b) the right hemispheres (bilateral Inferior Parietal Lobule, STG), and (c) frontal lobes (DLPFC) as compared to monolingual children.

**Fig. 4:**
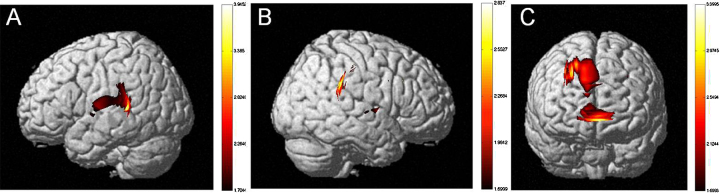
Neural activation of later-exposed bilingual children as compared to early-exposed bilingual children (*t*-statistic map from HbO, *p* = .05, corrected). Later-exposed bilingual children show more robust neural activation in (a) the left and (b) the right hemispheres (bilateral STG, right Inferior Parietal Lobule) and (c) frontal lobes (DLPFC, Frontopolar area) as compared to early-exposed bilingual children.

**Fig. 5:**
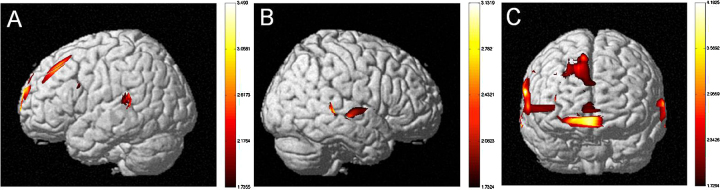
Neural activation of later-exposed bilingual children as compared to monolingual children (*t*-statistic map from HbO, *p* = .05, corrected). Later-exposed bilingual children show more robust neural activation in (a) the left and (b) the right hemispheres (bilateral STG), and (c) frontal lobes (DLPFC, Frontopolar area) as compared to monolingual children.

*Sentence Type*: The MLM revealed a significant difference between the more difficult SO versus OS sentence types (*F*(1,3715444) = 4.472, *p* < .05). HbO activation maps also revealed a significant difference between SO and OS sentences (see Fig. 2a). Greater neural activation was observed for SO as compared with OS sentences. *Language Group*: The MLM revealed a significant difference between monolinguals and bilinguals (*F*(1,1701) = 4.464, *p* < .05). HbO activation maps also revealed a significant difference between monolinguals and early-exposed bilinguals (see Fig. 3). Greater neural activation was observed for early-exposed bilinguals as compared with monolinguals. *Age of Second Language Exposure*: The MLM revealed a significant difference between early-exposed and later-exposed bilinguals (*F*(1,1701) = 4.464, *p* < .001). HbO activation maps also revealed a significant difference between monolinguals, early-exposed bilinguals, and later-exposed bilinguals. Greater neural activation was observed for later-exposed bilinguals as compared with early-exposed bilinguals (see Fig. 4) and for later-exposed bilingual as compared with monolinguals (see Fig. 5). We also observed a significant Language Group × Sentence Type (*F*(1,3715438) = 193.509, *p* < .001) and a significant Age of Second Language Exposure × Sentence Type interaction (*F*(1,3715438) = 142.814, *p* < .001).

##### 3.2.1.2 Adults

**Fig. 6:**
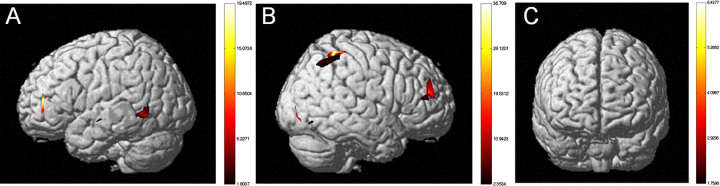
Neural activation of early-exposed bilingual adults as compared to monolingual adults (*t*-statistic map from HbO, *p* = .05, corrected). Bilingual adults show more robust neural activation in (a) the left (IFG, STG) and (b) the right hemispheres (IFG, Inferior Parietal Lobule) as compared to monolingual adults. (c) Bilingual and monolingual adults do not differ in frontal lobe activation.

*Sentence Type*: The MLM revealed a significant difference between the more difficult SO versus OS sentence types (*F*(1,1267769) = 115.882, *p* < .001). HbO activation maps also revealed a significant difference between SO and OS sentences (see Fig. 2b). Greater neural activation was observed for SO as compared with OS sentences. *Language Group*: The MLM revealed a significant difference between monolinguals and bilinguals (*F*(1,851) = 8.351, *p* < .01). HbO activation maps also revealed a significant difference between monolinguals and early-exposed bilinguals (see Fig. 6). Greater neural activation was observed for bilinguals as compared with monolinguals. We also observed a significant Language Group × Sentence Type interaction (*F*(1,1267769) = 7.373, *p* < .01).

#### 3.2.2 Regions of interest

##### 3.2.2.1 Left Inferior Frontal Gyrus

###### 3.2.2.1.1 Children

**Fig. 7:**
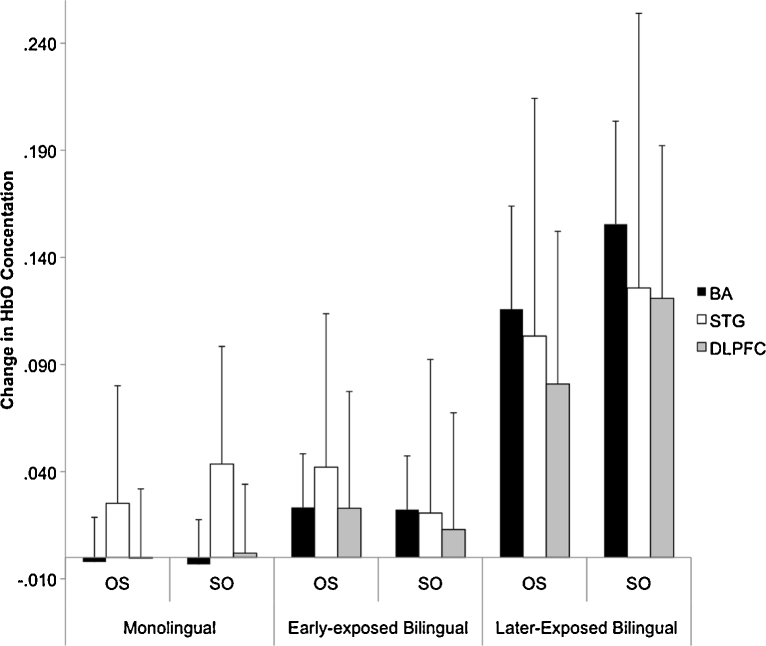
HbO concentration in bilateral Broca's area (BA), Superior Temporal Gyrus (STG) and Dorsolateral Prefrontal Cortex (DLPFC) for Object-Subject (OS) and Subject-Object (SO) sentences types across monolinguals, early- and later-exposed bilinguals. Later-exposed bilinguals show greater change in HbO concentration in bilateral Broca's Area (BA) and in the Dorsolateral Prefrontal Cortex (DLPFC) for both Object-Subject (OS) and Subject-Object (SO) sentences types relative to monolinguals (*p* < .05). Standard errors are indicated by bars.

*Sentence Type*: The MLM revealed a significant difference between the more difficult SO versus OS sentence types (*F*(1,157185) = 24.407, *p* < .001). *Language Group*: The MLM did not reveal a significant difference between monolinguals and bilinguals (*F*(1,70) = .180, *p* > .05). *Age of Second Language Exposure*: The MLM revealed a significant difference between early-exposed and later-exposed bilinguals (*F*(1,70) = 4.801, *p* < .05). We also observed a significant Age of Second Language Exposure × Sentence Type (*F*(1,157186) = 269.702, *p* < .001; see Fig. 7).

###### 3.2.2.1.2 Adults

*Sentence Type*: The MLM revealed a significant difference between the more difficult SO versus OS sentence types (*F*(1,44109) = 25.158, *p* < .001). *Language Group*: The MLM did not reveal a significant difference between monolinguals and bilinguals (*F*(1,31) = .655, *p* > .05).

We also observed a significant Language Group × Sentence Type interaction (*F*(1,44109) = 11.392, *p* < .01).

##### 3.2.2.2 Right Inferior Frontal Gyrus

###### 3.2.2.2.1 Children

*Sentence Type*: The MLM revealed a significant difference between the more difficult SO versus OS sentence types (*F*(1,131688) = 36.179, *p* < .001). *Language Group*: The MLM not reveal a significant difference between monolinguals and bilinguals (*F*(1,57) = .180, *p* > .05). *Age of Second Language Exposure*: The MLM did not revealed a significant difference between early-exposed and later-exposed bilinguals (*F*(1,57) = .007, *p* > .05). We also observed a significant Language Group × Sentence Type (*F*(1,131688) = 21.490, *p* < .001) and Age of Second Language Exposure × Sentence Type interaction (*F*(1,131688) = 178.531, *p* < .001; see Fig. 7).

###### 3.2.2.2.2 Adults

*Sentence Type*: The MLM revealed a significant difference between the more difficult SO versus OS sentence types (*F*(1,56322) = 125.458, *p* < .001). *Language Group*: The MLM did not reveal a significant difference between monolinguals and bilinguals (*F*(1,31) = .655, *p* > .05).

We also observed a significant Language Group × Sentence Type interaction (*F*(1,44109) = 11.392, *p* < .01), bilinguals showed greater neural activation as compared with monolinguals for SO sentence types.

##### 3.2.2.3 Left Superior Temporal Gyrus

###### 3.2.2.3.1 Children

*Sentence Type*: The MLM revealed a significant difference between the more difficult SO versus OS sentence types (*F*(1,125084) = 18.068, *p* < .001). *Language Group*: The MLM not reveal a significant difference between monolinguals and bilinguals (*F*(1,57) = .050, *p* > .05). *Age of Second Language Exposure*: The MLM revealed a significant difference between early-exposed and later-exposed bilinguals (*F*(1,57) = 8.992, *p* < .01). We also observed a significant Language Group × Sentence Type (*F*(1,125084) = 56.051, *p* < .001) and significant Age of Second Language Exposure × Sentence Type interaction (*F*(1,125084) = 53.865, *p* < .001; see Fig. 7).

###### 3.2.2.3.2 Adults

*Sentence Type:* The MLM revealed a significant difference between the more difficult SO versus OS sentence types (*F*(1,65128) = 124.052, *p* < .001). *Language Group*: The MLM did not reveal a significant difference between monolinguals and bilinguals (*F*(1,43) = .846, *p* > .05). We also observed a significant Language Group × Sentence Type interaction (*F*(1,65128) = 20.213, *p* < .01).

##### 3.2.2.4 Right Superior Temporal Gyrus

###### 3.2.2.4.1 Children

*Sentence Type*: The MLM revealed a significant difference between the more difficult SO versus OS sentence types (*F*(1,185108) = 27.417, *p* < .001). *Language Group*: The MLM revealed a significant difference between monolinguals and bilinguals (*F*(1,82) = 4.170, *p* < .05). *Age of Second Language Exposure*: The MLM revealed a significant difference between early-exposed and later-exposed bilinguals (*F*(1,82) = 4.556, *p* < .05). We also observed a significant Language Group × Sentence Type (*F*(1,185108) = 161.878, *p* < .001) and significant Age of Second Language Exposure × Sentence Type interaction (*F*(1,185108) = 38.024, *p* < .001; see Fig. 7).

###### 3.2.2.4.2 Adults

*Sentence Type*: The MLM did not reveal a significant difference between the more difficult SO versus OS sentence types (*F*(1,70921) = .122, *p* > .05). *Language Group*: The MLM revealed a marginally significant difference between monolinguals and bilinguals (*F*(1,46) = 3.145, *p* = .083). We also observed a significant Language Group × Sentence Type interaction (*F*(1,70921) = 30.599, *p* < .001).

##### 3.2.2.5 Dorsolateral Prefrontal Cortex

###### 3.2.2.5.1 Children

*Sentence Type*: The MLM revealed a significant difference between the more difficult SO versus OS sentence types (*F*(1,636222) = 7.983, *p* < .01). *Language Group*: The MLM did not reveal a significant difference between monolinguals and bilinguals (*F*(1,287) = 2.579, *p* > .05). *Age of Second Language Exposure*: The MLM revealed a significant difference between early-exposed and later-exposed bilinguals (*F*(1,287) = 20.121, *p* < .001). We also observed a significant Language Group × Sentence Type (*F*(1,636222) = 88.466, *p* < .001) and significant Age of Second Language Exposure × Sentence Type interaction (*F*(1,636222) = 18.180, *p* < .001; see Fig. 7).

###### 3.2.2.5.2 Adults

*Sentence Type*: The MLM revealed a significant difference between the more difficult SO versus OS sentence types (*F*(1,160885) = 20.666, *p* < .001). *Language Group*: The MLM did not reveal a significant difference between monolinguals and bilinguals (*F*(1,102) = 2.068, *p* > .05).

We also observed a significant Language Group × Sentence Type interaction (*F*(1,160885) = 182.348, *p* < .001).

##### 3.2.2.6 Control site (Primary Motor Cortex)

###### 3.2.2.6.1 Children

*Sentence Type*: The MLM did not reveal a significant difference between the more difficult SO versus OS sentence types (*F*(1,821388) = .000, *p* > .05). *Language Group*: The MLM did not reveal a significant difference between monolinguals and bilinguals (*F*(1,376) = .192, *p* > .05). *Age of Second Language Exposure*: The MLM did not reveal a significant difference between early-exposed and later-exposed bilinguals (*F*(1,376) = 2.553, *p* > .05).

###### 3.2.2.6.2 Adults

*Sentence Type*: The MLM did not reveal a significant difference between the more difficult SO versus OS sentence types (*F*(1,269369) = .451, *p* > .05). *Language Group*: The MLM did not reveal a significant difference between monolinguals and bilinguals (*F*(1,177) = 3.040, *p* > .05).

## 4 Discussion

The present study seeks insights into the nature of language processing in the developing bilingual brain. Does bilingual language experience in early life change the functional organization of the brain that supports language and aspects of higher cognitive processing? We were especially interested to learn whether the age of first bilingual language exposure can yield neural changes in classic language areas (LIFG, STG) and cognitive areas (e.g., DLPFC) during syntactic processing. Do young bilinguals recruit neural resources supporting sentence processing in a manner similar to or different from adult bilinguals? If so, does this increased neural activation in classic language areas depend on the age of bilingual exposure (early versus later)? These questions allowed us to gain new insights into whether the developing brain also exhibits the propensity for expanded neural recruitment of classic language tissue that has been observed in the adult bilingual brain, called “the neural signature of bilingualism” (Kovelman et al., 2008a).

Using a sentence judgment task consisting of sentences varying in syntactic and semantic complexity (including “default” OS sentences, and more difficult SO sentences), as predicted, our behavioral data revealed greater inaccuracy and reaction times when children and adults judged SO as compared to OS sentence types in English (Kovelman et al., 2008a, Caplan et al., 2008a, Caplan et al., 2008b, Caplan et al., 2003, Caplan et al., 2000, Caplan et al., 1998, Chen et al., 2006, Stromswold et al., 1996). Here, we observed greater neural recruitment of the bilateral IFG for SO as compared with OS sentence types among adults, corroborating previous research (Kovelman et al., 2008a, Caplan et al., 2008a, Caplan et al., 2008b, Caplan et al., 2003, Caplan et al., 2000, Caplan et al., 1998; Chen et al., 2006; Stromswold et al., 1996). Children also showed greater neural recruitment of the bilateral IFG for SO as compared with OS sentence types. However, while adults showed more robust activation for SO sentence types in the IFG, children showed more robust activation for SO sentence types in the MTG. While this is an intriguing developmental difference between child and adult, in itself, the activation pattern observed is not wholly unexpected. The STG is involved in processing complex sentences, particularly in integrating semantic and syntactic information (Friederici et al., 2009), the MTG is also involved in sentence processing (Vandenberghe et al., 2002, Newman et al., 2001).

Why might we see the differences in neural activation between child and adult? To explain why the IFG is more active in the adult and the MTG is more active in the child, we offer the observation that maturational differences over time have been observed elsewhere in the literature, and, thereby, by analogy, rend the present neural pattern well motivated by typical brain developmental changes. For example, findings reviewed in Friederici and Gierhan (2013) identify neural pathways that connect the STG with the IFG. Following from this, we offer that the maturation of these or related neural pathways may contribute to the present finding showing developmental differences between temporal cortex (here, specifically the MTG) in children, and the IFG in adults. Other studies show intriguing developmental changes as well. The neural activation in left inferior parietal, left superior temporal and right temporal regions have been found to decline with age (Devous et al., 2006), while activation in the LIFG has been found to increase with age (Moore-Parks et al., 2010). Adults’ greater LIFG activation might be related to enhanced ‘top-down’ control that is involved in making a plausibility judgment about a syntactically complex sentence (Moore-Parks et al., 2010).

Regarding differences among our monolingual and bilingual groups, behavioral data revealed similar reaction times and accuracy across the three groups (monolinguals, early-exposed bilinguals, later-exposed bilinguals). Although bilingual children demonstrated better performance on the sentence judgment task relative to their monolingual peers, this effect was not statistically significant (e.g., accuracy rates of 56.1% and 74.6% for SO and OS sentences respectively among early-exposed bilingual, as compared with 49.5% and 63.5% among monolingual children). While the behavioral data did not reveal any group differences in sentence processing, only the neuroimaging data revealed differences in neural recruitment among monolinguals, early-exposed bilinguals and later-exposed bilinguals—those that carry important theoretical implications regarding contemporary questions about the nature of the bilingual brain. It is clear that this fascinating finding warrants more formal study, but one hypothesis (among several) that we would test is that the English shows greater activation in later exposed bilinguals because there may be stronger linguistic co-activation between the earlier-exposed and the later-exposed language.

First, our bilingual adult controls corroborated the finding that adult bilinguals recruit a greater extent and variability of classic language areas as compared to monolingual adults (Kovelman et al., 2008a, Kovelman et al., 2008c). Here our SPM and MLM analyses both revealed greater neural recruitment of the STG among bilinguals as compared with monolinguals. Yet one of the most surprising findings of the study were the neural differences that we observed in even the youngest bilingual child, as compared with her monolingual peer. Be she an early-exposed or a later-exposed bilingual child, we found greater neural activity in classic left hemisphere language tissue, as well as their right hemisphere homologues, in our bilingual groups as compared to monolinguals. Despite the fact that the young brain has yet to undergo substantial maturational changes that will facilitate language processing, early life bilingual experience has modified the extent to which classic language tissue (LIFG, Broca's, STG, and their right hemisphere homologues) is recruited for aspects of sentence processing. That this “neural signature” of bilingualism was also present in the *developing* brain suggests that the neural tissue underlying language processing, the LIFG and STG, may be modified as a result of bilingual language experience *before* it has matured and indicates that bilingualism abides by principles of “Sensitive Period Hypothesis” (Lenneberg, 1967, Newport, 1990).

A novel design feature of this study is that we examined young early-exposed bilingual children (ages birth to age 3 years) as compare to later-exposed bilingual children (ages 4–6 years)—with particular attention given to the nature of language processing in their first/earliest exposed language (in this case, English). While it is understood that language proficiency can impact neural processing (Marian et al., 2003, Hahne and Friederici, 2001, Wartenburger et al., 2003, Chee et al., 2004, Perani et al., 2003, Nauchi and Sakai, 2009), we held constant language proficiency by comparing all bilingual groups’ earliest exposed language (English) with English monolingual controls. Here we found that early-exposed bilingual children showed a different pattern of neural activation as compared to later-exposed bilingual children. Later-exposed bilingual children showed greater neural recruitment of classic language areas (LIFG, Broca's Area, STG) and cognitive-general areas (DLPFC) as compared to early-exposed bilinguals. This observation implies that the age of first bilingual language exposure has the potential to modify the brain's developmental trajectory supporting language processing. Previous research has found differences in behavioral and neural activation for early-exposed bilinguals and later-exposed bilinguals in their second language (Marian et al., 2003, Hahne and Friederici, 2001, Wartenburger et al., 2003, Chee et al., 2004, Perani et al., 2003, Nauchi and Sakai, 2009). The field has attributed these differences to the later age of acquisition and/or to the lower proficiency levels in the second language. However, the bilinguals in the present study performed the task in their earliest exposed language, which they had all acquired from birth, used regularly, and were highly proficient in. Thus, we were able to directly assess whether the age of bilingual language exposure would modify the neural organization supporting language processing in the *first language* of all participants. This was precisely what we observed.

The present findings also revealed new insights into the neural resources that facilitate language processing in the bilingual brain. Is bilingualism largely a cognitive-general process, whereby dual language processing places increased demands on higher executive functions? Or is bilingualism largely supported by changes to the brain's classic language tissue? Here, we observed robust recruitment of classic language areas in bilingual children (and adults) relative to monolinguals, providing support for the hypothesis that the fundamental nature of language in the bilingual brain is supported by changes to classic language tissue. To be sure, we observed greatest increased recruitment of classic cognitive-general tissue (DLPFC) among later-exposed bilinguals. Thus, the neural resources (language-specific, or cognitive-general) that support the processing of two different languages within one brain are modifiable by the age of first bilingual exposure.

## 5 Conclusion

Compelling evidence was observed in support of the hypothesis that bilingualism imparts fundamental changes to classic language areas as opposed to alterations to brain regions governing classic higher cognitive executive functions. However, the age of first bilingual exposure does matter. With respect to the later-exposed bilinguals, we observed the neural recruitment of both increased cognitive-general and language-specific resources as compared to monolinguals and early-exposed bilinguals. These results reveal new information about the potential extent and variability of language-dedicated neural tissue and its developmental trajectory, and how this may be modified through experience when a child is exposed to one or two languages at different points in development. Dual language exposure appears to have an impact on how the bilingual brain engages the regions and areas that underlie human language and the degree of activation in these areas. Taken together, and most fascinating to us, is this: The bilingual language user may provide a powerful new window into the human language processing potential that is not fully recruited (engaged) in monolinguals. The findings from the bilingual brain lead us to a tantalizing view of the fullest biological extent of the neural tissue underlying language, which may be exploited in the bilingual and possibly lost in the monolingual.

How much can bilingual exposure modify the neural organization for language, whether multilingualism, relative to bilingualism, yields more variable neural recruitment, and at what point in language development is the brain most susceptible to experience-based changes, all demand further investigation. Yet one observation stands strong: The bilingual's robust recruitment of classic language areas may constitute the underlying neural systems that give rise to the language and reading advantages observed among bilingual children relative to their monolingual peers (Kovelman et al., 2008b).
